# Consent and Autonomy in the Genomics Era

**DOI:** 10.1007/s40142-019-00164-9

**Published:** 2019-05-02

**Authors:** Rachel Horton, Anneke Lucassen

**Affiliations:** 1 Clinical Ethics and Law at Southampton (CELS), Faculty of Medicine, University of Southampton, Centre for Cancer Immunology, Southampton General Hospital, Southampton, SO16 6YD UK; 2 Wessex Clinical Genetics Service, Princess Anne Hospital, Southampton, SO16 5YA UK

**Keywords:** Ethics, Consent, Autonomy, Genomics, Broad consent, Relational autonomy

## Abstract

**Purpose of Review:**

Genomic tests offer increased opportunity for diagnosis, but their outputs are often uncertain and complex; results may need to be revised and/or may not be relevant until some future time. We discuss the challenges that this presents for consent and autonomy.

**Recent Findings:**

Popular discourse around genomic testing tends to be strongly deterministic and optimistic, yet many findings from genomic tests are uncertain or unclear. Clinical conversations need to anticipate and potentially challenge unrealistic expectations of what a genomic test can deliver in order to enhance autonomy and ensure that consent to genomic testing is valid.

**Summary:**

We conclude that ‘fully informed’ consent is often not possible in the context of genomic testing, but that an open-ended approach is appropriate. We consider that such broad consent can only work if located within systems or organisations that are trustworthy and that have measures in place to ensure that such open-ended agreements are not abused. We suggest that a relational concept of autonomy has benefits in encouraging focus on the networks and relationships that allow decision making to flourish.

## Introduction

Genomic tests can give a range of different outputs: a few genomic results alter treatment in a life-changing way; some provide an explanation for an illness and options for the future; several raise more questions than answers; some find unexpected or unwelcome information; and many provide nothing useful at all. Some find nothing for now, but ongoing interpretation may find useful information in the future. Consent processes therefore need to encompass a wide range of outcomes and uncertainties, and prepare people for very contrasting possibilities. Further complexity arises in that genomic testing for one person may reveal information of relevance to their family members, or interpretation of one person’s genetic information may require their relatives to participate in testing—who can give or withhold consent to such a test?

Genome sequencing identifies over four million variants per person [1], which need distillation to arrive at a meaningful output. Arriving at a genomic ‘result’ will depend on what filters are applied to the raw data, which in turn will depend on what clinical questions are asked. The (mostly) fixed nature of a person’s variants over their lifetime and the strongly deterministic popular discourse surrounding genomics [2••] tempt us to imagine that we might be able to work out what each variant does and somehow add this knowledge up to give useful results. However, the variants highlighted in the course of genomic testing will often have very little effect in isolation, and their context, both in terms of the genetic background against which they operate, and other factors such as environmental exposures, may determine whether their presence manifests in disease. This creates much scope for misinterpretation of a genome output, as the contextual factors that influence the clinical consequences of particular variants are often poorly understood and underappreciated. Our understanding of the clinical significance of any given variant will fluctuate with respect to lots of parameters, including scientific certainty and the current state of scientific knowledge, the age of the person being tested, their context in a biological family, and their medical history [3, 4•, 5].

Before next-generation sequencing technologies became routinely available, clinicians would carefully select genes to look at which they thought might explain a patient’s personal or family history of disease. If a rare variant that looked like it would disrupt a gene product was found in this context, it was quite likely to explain the condition. Prior probabilities in the genomic era are radically different: everyone has around 100,000 rare variants [1], most of which will have essentially no impact on health. There is increasing acceptance that every variant identified via genomic testing should be considered innocent until proven guilty [6], but whilst this stance minimises harm from overzealous interpretation of genomic tests, it continues to force a binary appraisal, where variants are either ‘benign’ or ‘disease-causing’. This risks adding fuel to the idea that the presence of particular variants inexorably leads to specific diseases, and makes it difficult to appropriately conceptualise subtle risks. Population studies should undermine our confidence in the predictive ability of even ‘well-known’ genetic variants. For example, the R114W variant in *HNF4A*, which was thought to have a penetrance of 75% by age 40 based on studying families with maturity-onset diabetes of the young (MODY) [7], was found to have a penetrance of ~ 10% by 40 years in people from UK Biobank [8••]. Similarly, recent research indicates that less than a third of people with ‘disease-causing’ variants in *SDHB* will develop a clinically apparent paraganglioma or phaeochromocytoma by age 80 [9]; initial studies had indicated 50% penetrance by 35 years [10].

In summary, genomic investigations are complex, and might produce multiple results over time as different questions are asked of the same raw data, or different knowledge is brought to bear in the interpretation process. It is difficult to apply notions of ‘informed consent’ to a situation where the possible outcomes are so unknown, both by virtue of the individuality of the genomic data inputted, but also due to the complexity of navigating through that data to a ‘result’.

## Are We Achieving Valid Consent in the Genomic Setting?

The practice of consent in medicine has largely evolved around simpler scenarios, for example whether or not to have an operation, or whether or not to start a medication. The risks are often more easily foreseen, and the possible outcomes are clearer. In trying to shoehorn notions of consent derived from these situations into the scenario of genomic testing, we run into various difficulties.

In legal terms, for consent to be valid, the patient needs to be competent; they need to be appropriately informed; and their consent needs to be voluntarily given [11]. In the arena of genomic testing, all of these elements are arguably in doubt. For a person to be competent to make a particular decision, they need to be able to understand, retain, and weigh the information necessary to decide, and communicate their wishes [12]. For genomic tests, it is doubtful whether anyone can really fulfil these criteria—the ramifications of genomic testing may be unpredictable even for people who know the field well. For example, Watson was one of the first people to have his genome sequenced and chose to have his *APOE* information redacted (as he preferred not to know if he had an *APOE* genotype associated with an increased chance of developing Alzheimer’s disease). However, from the information that was put in the public domain, it was still possible to infer Watson’s *APOE* genotype [13]: world experts were being tested and doing the testing but this issue still arose. Watson’s *APOE* information was insufficiently protected because all parties involved were at that time unaware that this could be inferred from linked markers in the public domain. In consent conversations, healthcare professionals are charged to give patients the information that they want or need to make a particular decision [12], but in the context of genomics, we should not assume that anyone can anticipate all the likely or serious potential consequences of testing.

‘Appropriately informing’ a person making a decision about genomic testing is a challenging task. For example, research indicates that several 100,000 Genomes Project participants have an inaccurate recollection of whether or not they have chosen to know about health risks unrelated to the condition that led to them joining the project. Whatever they had chosen, many people assumed that they would be told if ‘something important’ was found [14]. Despite a detailed consent process, what patients thought they agreed was sometimes different to what their clinicians thought they had agreed. If consent is judged purely on understanding and retention of relevant information, it did not work well for these patients.

Often, participation in genomic testing has wider consequences—for example, in accessing clinical testing via the 100,000 Genomes Project, participants also agree to make their data available for use by research and industry (with various safeguards). Some people want a clinical diagnosis but might be reticent about data sharing—does making the two a ‘package deal’ undermine the voluntariness of their consent? We can argue that a genome cannot be interpreted in isolation: it relies on comparison to ‘normal’ controls, and databases of ‘abnormal’ disease-causing variants, hence data sharing is an integral part of the interpretation process [15]. We can also compare it to situations where achieving one desired outcome requires compliance with other processes, such as needing to show your passport to travel abroad. However, a genomic diagnosis would make a great difference to a number of people and families, and requiring people to give their data to industry in order to pursue a desperately needed diagnosis could be portrayed as coercive (perhaps especially since over half will not get a diagnosis via genomic testing, at least in the short term).

## How Can We Prepare Robust Consent Processes for the Genomic Era?

The variety of possible outcomes from genomic tests, and the illusion that navigation towards such outcomes is straightforward, gives an impression that genomic testing should dovetail nicely with the politically popular stance that more choice is always good [16]. However, the complexity of genomic test interpretation, which means that patient choice can be difficult to straightforwardly honour, risks feeding into fears that in exercising their judgement as to what constitutes a clinically meaningful result, clinicians and scientists are being paternalistic. ‘Binning’ models have been suggested to guide return of results to research participants based on factors such as clinical utility or reproductive significance [17], but many genetic variants are difficult to neatly classify as their effects may be heavily context dependent [8••], and different people may attribute different significance to the same numerical risk [18].

The idea of ‘broad consent’ has gained traction in circumstances where, by virtue of the uncertainty and diversity of possible interventions or outcomes, people can consent based on agreement with general principles that govern what will be done, without knowing specific details of what will actually end up being done. People contributing to biobanks provide consent under such a model, essentially choosing to delegate decisions about what future research will be done with their samples based on some knowledge of what the decision-making process would involve [19]. Participation in genomic testing has some similarity. For example, participants in the 100,000 Genomes Project have a choice whether or not to receive ‘additional findings’ (genetic information with screening/treatment implications that is unrelated to the condition that led to them joining the project), but the exact ‘additional findings’ looked for may alter over time [20].

Arguments relating to biobank participants have convincingly made the case that broad consent can be informed—it does not have to be ethically inferior to specific consent [19]. However, diagnostic genomic testing has various differences to participation in a biobank: people’s motivations and expectations will be different. These boundaries are increasingly blurred by hybrid clinical research initiatives such as the Deciphering Developmental Disorders (DDD) project and the 100,000 Genomes Project, but in these cases, a patient or family is usually seeking an explanation for an existing condition, perhaps taking a ‘research’ avenue because standard clinical testing has not been fruitful.

Whilst altruistic motives may be important, participation in diagnostic projects such as DDD or the 100,000 Genomes Project may differ from biobanks as engagement with the project is more reciprocal—data might be given with the hope and/or expectation that useful information will be communicated in return. We think that broad consent can still operate here, but the increased potential for ongoing engagement between a person, and data derived from their sample, may mean that a laissez-faire delegation of decision making sits less comfortably than for biobanks, where people may perceive themselves as donating a sample that goes into the ether never to be heard of again.

In diagnostic enterprises, people give data wanting answers for themselves or their family; the personal impact of delegated decision-making processes is far greater on them than it would be on biobank participants. They may understandably want more information as to what is happening to their sample, but it is often unclear how best to honour this, as increasing the quantity of findings communicated may compromise the quality, with a risk that important findings will be subsumed within a mass of variants of dubious clinical significance. Research indicates that many people feel unhappy that decisions about disclosure of uncertain variants may be made without them [21], although perhaps this reflects a concern that such decisions may involve clinicians and scientists debating whether to hide information about patients, rather than discussing whether the presence of particular variants constitutes information in any clinically helpful sense [22]. Correspondingly, clinicians and scientists may be apprehensive that patients might attribute too much significance to variants identified via genetic or genomic testing [23, 24], and be harmed as a consequence of overly deterministic reactions [25].

One response to these issues is to ensure that participants have a voice in these decision-making processes. For example, the 100,000 Genomes Project has a Participant Panel that advises Genomics England on decisions relating to data use. Individual participants cannot ultimately decide how their own data is interpreted, but they can become involved in governance processes that guide how their data might be interpreted, and if dissatisfied with this, they can withdraw from testing. Such processes, albeit unintentionally, are perhaps most likely to engage well-educated people who are already interested in the topic. Input and perspectives from people with different educational backgrounds, or people who are unaware of the potential relevance to themselves, may be more difficult to elicit [26]. In pointing out that efforts to ensure participant involvement will inevitably have imperfections, we do not mean to imply that they lack value. On the contrary, we think that participant guidance of decision-making processes is very important, and highlight concerns regarding representation as something that might benefit from more focus rather than an issue that should undermine such enterprises.

One person’s consent may have implications for a breadth of others: in participating in genomic testing, a person may find information of relevance to their biological relatives. Sometimes this may be overtly related to health and unambiguous, for example detecting the same rare disease–causing *BRCA1* variant in two cousins with young-onset breast cancer will in essence find the same variant in the parents who connect them. Sometimes the relevance to health will be more uncertain or context-dependent, for example a finding of misattributed paternity in a child with developmental delay [27]. Typically, we only ask the consent of the person providing a DNA sample for testing (or their representative if under-age or lacking capacity). Others in their family who might be diagnosed through their test are not asked, although in certain circumstances this possibility would be explored at length as part of the consent process.

A person’s ‘genomic result’ is often inextricably connected to that of their biological relatives, regardless of whether this is comfortable or convenient from a social perspective. Notions of consent that conceptualise patients as entirely separate from their biological family are therefore liable to fall short. We consider that heritable genetic information should be confidential to families, not individuals (though the personal consequences for a given person of having a particular variant should be confidential to them alone) [28]. It is important to explore this in advance of genomic testing, partly to pre-empt the very rare situations where patients are unwilling to share health-relevant genetic information with their biological relatives, but more commonly, to consider how such communication might be supported if necessary. In offering genomic testing to one person, we have to be aware that potentially we are testing other people too, without consent.

A further aspect that needs particularly careful handling is the possibility for genomic test interpretation to evolve or be overturned as scientific knowledge advances [29, 30••]. Consent discussions need to tread a delicate balance between leaving things open such that patients are aware that things might change, and not engendering an expectation that genomic data will routinely be examined again and patients will be updated, if no such mechanism is in place to ensure this. There is often lack of clarity about who might be responsible for recontacting patients if new information arises, or has the potential to arise, from old genetic or genomic data [31•, 32]. Any responsibility seems to lie somewhere between patients and health professionals, with a likely upshot being that in some cases no-one sees it as their responsibility, and so recontact never happens.

Given the complexity and evolving nature of genomic test interpretation, we argue that consent for genomic testing may be helpfully conceptualised as an ongoing conversation, where aspects of the discussion are revisited as necessary over the course of a person’s life. However, rather than rely on (albeit ongoing) consent to ‘do all the work’, we consider it will need to be situated within a trustworthy system, where patients can have confidence that their genomic data will be treated with care, and that their preferences will be accommodated as much as possible without the need to specify up-front exactly what will be sought or found [33]. Finding an appropriate balance between re-opening discussions where necessary, and intruding unexpectedly in people’s lives to ask potentially unwelcome questions, will clearly be difficult. However, we think that this approach would get us closer to valid consent, than treating consent as a one-off rubber-stamping event, and approximating answers to any and every subsequent question by looking at whatever form the patient filled in on that day. The need to move away from a tick-box mentality in consent conversations is reflected in the upcoming Consent and Confidentiality in Genomic Medicine guidance, which offers a ‘Record of Discussions’ form as opposed to a consent form for genomic testing [34].

## What Autonomy Is Possible in Genomic Testing?

If specific consent is not possible, and test results may inevitably impinge on others beyond the person providing a DNA sample, what does this mean for autonomy? If we consider autonomy to be the self-rule of independent people—their right to determine what happens to them—then various aspects of genomic testing threaten to scupper it. Most people cannot sequence and interpret their own genome, so a person provides a sample for testing, perhaps with some guidance as to what sort of information they might want returned from it, but the interrogation of that sample will involve multiple decisions where they cannot give personal input. For example, what databases should be filtered against in order to identify candidate variants? What threshold of scientific certainty must be reached before a variant is communicated as a possible result? Clearly, some threshold is necessary, as communicating 100,000 rare variants to each person would not be useful. This distancing of decisions from the patients to whom they relate could be seen as at odds with respecting their autonomy, although if we consider genomic testing as a procedure, such practices are common to many areas of medicine. For example, a person consenting to an appendicectomy would not usually expect to be consulted on exactly which brand of surgical instruments and sutures to use in the process of removing the appendix.

A more difficult issue is that via a lens of individualistic autonomy, testing any one person may rob their relatives of their autonomy, as information may be found of relevance to them that they did not decide (or perhaps want) to pursue. However, refusing to provide a genomic test to someone on the basis that it may damage the autonomy of others undermines that person’s own autonomy. Individualistic concepts of autonomy run aground here: in treating each person as entirely independent of others, we risk drawing irreconcilable conclusions.

Relational autonomy may offer a more useful way to conceptualise autonomy in the genomic era. This considers a person as situated within a network of others, arguing that relationships and social surroundings are central in allowing people to develop a sense of self, and a capacity for self-determination [35]. In choosing to pursue genomic testing, generating results, and reacting to them, multiple people make interdependent decisions. A relational conception of autonomy is more able to reflect and accommodate this complexity: networks of trust and care guide choices, and room is left for shared decision making [36]. Relational autonomy maps more closely to the real world messiness of people and their decisions to undergo genomic tests, than conceptualising each person as an independent agent making decisions in a social vacuum. But in doing so, it draws our focus to the networks that allow decision making to flourish—are these prepared for the genomic era?

One key element that we think requires attention is the societal discourse around genomics—currently, this errs towards being strongly deterministic and enthusiastic about the benefits of testing [2••], risking opening up a gulf between expectation and reality for people receiving genomic results. Genomic results are complex, unpredictable, or heavily context dependent, but this nuance is often missing from information in the public domain. This issue is perhaps intensified by the advertising of direct-to-consumer genetic testing companies [37], who have a commercial interest in presenting genetic tests as clearly predictive, conveying information and thereby conferring power. In reality, genetic test results often do not appear to change behaviour [38]; polygenic risk scores, whilst helpful in understanding causation, are unable to usefully predict disease [39]; and the predictive ability of ‘disease-causing’ variants may be heavily dependent on the context in which they were ascertained [8••].

Evidence suggests that with increasing knowledge of these issues, people tend to make more conservative choices as to what they might want communicated from genome sequencing [40, 41]: the perceived value of genomic information may decline with increasing familiarity with the field. However, this must be juxtaposed against research that indicates that many people have a strong desire to receive even uncertain findings from genetic and genomic tests [42]. Negotiating appropriate thresholds for communication of genomic information is an ongoing challenge. The substantial discrepancy between what popular discourse leads us to expect from genomics, and what genomics might actually be capable of providing, needs to be tackled. Part of the problem is that naturally we are drawn towards celebrating the successes of genomic medicine, and political and funding systems strongly incentivise this focus. The many people for whom genomic testing is (at least at present) unhelpful are not given comparable airtime, although organisations such as Syndromes Without A Name make great efforts to ensure that their voices are heard. In creating an environment that supports people’s abilities to make meaningful choices regarding genomic testing, we need to ensure that public discussions in this area appropriately reflect the realities of what it is likely to provide.

A further element that needs attention in order to allow decision making to flourish is how best to support the trustworthiness of institutions where genomic test interpretation takes place [33]. The complexity and uncertainty of genomic results means that it is sometimes hard to know how best to respect an ostensibly clear-cut choice—for example, people might mean different things by a ‘serious’ condition, or an ‘actionable’ finding [43]. Unambiguous choice is often an illusion in the context of genomics, and in avoiding confronting this issue, we risk generating a loss of trust, where people feel they were asked to make unrealistic choices.

The question then arises of how to create a trustworthy system: transparency is frequently advocated but the complexity and specialist nature of genomic interpretation means that efforts at transparency risk appearing token or evasive, perhaps undermining the very trust that they try to create [44]. Perhaps, we need to acknowledge that genomic interpretation involves multiple decisions where patients cannot have direct input, and focus on how patient preference might meaningfully influence the scientists and clinicians making those decisions, rather than ignoring such decisions because their necessity is unfashionable.

## Conclusions

Targeted genetic tests have always had consequences for others beyond the person being tested, and have sometimes generated uncertain results. Genomic tests amplify this uncertainty exponentially, and add a further dimension of heightened complexity and unpredictability. As such, genomic tests present a challenge to traditional notions of consent and autonomy: how can a person consent to a process where the potential outcomes are so unknown and unpredictable, and how can a person independently determine how their genome is tested when the interests of their biological relatives may be inextricably dependent on their decisions? We argue that our concepts of consent and autonomy need to expand to be fit for purpose in the genomics era. In particular, we suggest that consent for genomic testing needs to be considered both as broader than specific consent as well as part of an ongoing conversation with room to open up further discussion where necessary, rather than being thought of as a time-locked event. We also argue that relational concepts of autonomy offer a more fruitful way to engage with the realities of genomic testing. On a biological level, a person shares their genetic code with their close relatives, and on a social level, a person choosing to undergo genomic testing engages in a process that requires others to use their experience and skill to interpret that individual’s genetic code. Considering how we can strengthen the networks and relationships that facilitate choices regarding genomic testing is an important aspect of enhancing autonomy, and a pre-requisite of ethical preparedness for the genomic era.
